# The Diminishing Mortality from Infectious Diseases

**Published:** 1898-01-15

**Authors:** 


### THE DIMINISHING MORTALITY FROM INFECTIOUS DISEASES.

An interesting paper by Dr. William C. Deming in the New York *Medical Record* shows the diminution which has taken place in the prevalence of infectious diseases during recent years. The facts which he brings forward have been collected from various sources and are of themselves of no great novelty. He has, however, put them together in a very striking way, and by the aid of charts has made it plain that from some cause or another a wide-spread influence is at work under which the prevalence of many infectious diseases is undergoing a decline. Whether we take phthisis, typhoid fever, or scarlet fever, and whether we trace their course in Massachusetts, in England, in Glasgow, in Paris, or in New York, we find a diminution in their mortality compared with what they caused about thirty years ago. The interesting question is how far we shall be right in attributing this to the spread of sanitary knowledge and to the largely increased expenditure on sanitary objects, which have been so marked a feature of the past quarter of a century; or, on the other hand, how far the improvement is due to some unknown influence in accordance with which these diseases tend to run in long cycles of twenty or thirty years. We do not think that, as things stand at present we have data enough to enable us to answer this question. Our statistics do not go back far enough, and even now, while we know something about mortality, we know nothing about the prevalence of many infectious diseases. There is, however, a good deal of evidence to show that some of these diseases, diphtheria, for example, have always run in long cycles. It behoves us, then, not to be too confident that all the improvement in the death-rates from fevers are entirely owing to our own endeavours. Especially important is it that we should not jump to the conclusion that individual methods, such, for example, as the hospital isolation of infectious diseases, has been the cause of their lessened mortality, for an equal improvement seems to have occurred in places where isolation has not been practised. Still, on the other hand, there is a good deal to show that, taking sanitation in its larger sense as covering all that improves the conditions of life, sanitary improvement has had a great deal to do with the prevention of death from infectious diseases. What is most marked, in fact, in the curves of recent years is not merely the lowered mortality (which, of course, might be due to cyclical variation), but the greatly lessened range of epidemic fluctuation. The tendency to cyclic variation is diminishing, and the prevalence of each disease is tending towards a common point.

